# Health profile of adult special immigrant visa holders arriving from Iraq and Afghanistan to the United States, 2009–2017: A cross-sectional analysis

**DOI:** 10.1371/journal.pmed.1003118

**Published:** 2020-05-13

**Authors:** Gayathri S. Kumar, Simone S. Wien, Christina R. Phares, Walid Slim, Heather M. Burke, Emily S. Jentes

**Affiliations:** 1 Immigrant, Refugee and Migrant Health Branch, Division of Global Migration and Quarantine, Centers for Disease Control and Prevention, Atlanta, Georgia, United States of America; 2 Oak Ridge Institute for Science and Education, Oak Ridge, Tennessee, United States of America; 3 Migration Health Division, International Organization for Migration, Erbil, Iraq; 4 Migration Health Division, International Organization for Migration, Amman, Jordan; Johns Hopkins University Bloomberg School of Public Health, UNITED STATES

## Abstract

**Background:**

Between 2,000 and 19,000 Special Immigrant Visa (SIV) holders (SIVH) from Iraq and Afghanistan resettle in the United States annually. Despite the increase in SIV admissions to the US over recent years, little is known about the health conditions in SIV populations. We assessed the burden of select communicable and noncommunicable diseases (NCDs) in SIV adults to guide recommendations to clinicians in the US.

**Methods and findings:**

We analyzed overseas medical exam data in Centers for Disease Control and Prevention’s (CDC) Electronic Disease Notification system (EDN) for 19,167 SIV Iraqi and Afghan adults who resettled to the US from April 2009 through December 2017 in this cross-sectional analysis. We describe demographic characteristics, tuberculosis screening results, self-reported NCDs, and risk factors for NCDs (such as obesity and tobacco use). In our data set, most SIVH were male (Iraqi: 59.7%; Afghan: 54.7%) and aged 18–44 (Iraqi: 86.3%; Afghan: 95.6%). About 2.3% of Afghan SIVH and 1.1% of Iraqi SIVH had a tuberculosis condition. About 0.3% of all SIVH reported having chronic hepatitis. Among all SIVH, 56.5% were overweight or had obesity, 2.4% reported hypertension, 1.1% reported diabetes, and 19.4% reported current or previous tobacco use. Iraqi SIVH were 3.7 times more likely to have obesity (95% CI: 3.4–4.0), 2.5 times more likely to report diabetes (95% CI: 1.7–3.5), and 2.5 times more likely to be current or former smokers (95% CI: 2.3–2.7) than Afghan SIVH. Limitations include the inability to obtain all SIVH records, self-reported medical history of NCDs, and the underdiagnosis of NCDs such as hypertension and diabetes because formal laboratory testing for NCDs is not used during overseas medical exams.

**Conclusion:**

In this analysis, we found that 56.5% of all SIVH were overweight or had obesity, 2.4% reported hypertension, 1.1% reported diabetes, and 19.4% reported current or previous tobacco use. In general, Iraqi SIVH were more likely to have obesity, diabetes, and be current or former smokers than Afghan SIVH. State public health agencies and clinicians doing domestic screening examinations of SIVH should consider screening for obesity—as per the CDC’s Guidelines for the US Domestic Medical Examination for Newly Arriving Refugees—and smoking and, if appropriate, referral to weight management and smoking cessation services. US clinicians can consider screening for other NCDs at the domestic screening examination. Future studies can explore the health profile of SIV populations, including the prevalence of mental health conditions, after integration into the US.

## Abstract 2

Gayathri Kumar and colleagues report the disease burden in adult special immigrant visa holders to guide recommendations to clinicians in the United States.

## Author summary

**Why was this analysis done?:**

- Between 2,000 and 19,000 Special Immigrant Visa holders (SIVH) from Iraq and Afghanistan have resettled in the United States annually since 2008.
- Per the Immigration and Nationality Act, SIVH, like other immigrants and refugees, must be examined by a physician before arriving in the US. Results of these overseas examinations are transmitted by the Centers for Disease Control and Prevention (CDC) to US state and local health departments via CDC’s Electronic Disease Notification system (EDN).
- Increasing provider knowledge about the health conditions most commonly encountered in SIVH as well as any differences in health conditions between SIVH from Iraq and Afghanistan may facilitate diagnostic screening, examination, and referrals to additional healthcare providers in the US.
- Information about the health of SIV populations is limited and would be beneficial for US clinicians who see SIVH in their clinics.

**What did the researchers do and find?:**

- In this cross-sectional analysis, we analyzed overseas medical exam data in CDC’s EDN for 19,167 SIV Iraqi and Afghan adults who resettled to the United States from April 2009 through December 2017.
- Among all SIVH, 56.5% were overweight or had obesity, 2.4% reported hypertension, 1.1% reported diabetes, and 19.4% reported current or previous tobacco use.
- In general, Iraqi SIVH were more likely to have obesity, diabetes, and be current or former smokers than Afghan SIVH.

**What do these findings mean?:**

- State public health agencies and clinicians screening SIVH should consider screening for diabetes among those with risk factors and prompt referral and management of obesity, hypertension, and smoking.
- Behavioral risk factor counseling and referral to culturally appropriate chronic disease prevention programs can be initiated at screening visits and subsequently reemphasized with primary care providers and other healthcare professionals.
- Limitations include the inability to obtain all SIVH records, self-reported medical history of NCDs, and underdiagnosis of NCDs such as hypertension and diabetes because formal laboratory testing for NCDs is not used during overseas medical exams.

## Introduction

The United States (US) resettles tens of thousands of refugees annually from around the world [1]. In addition, between 2,000 and 19,000 Special Immigrant Visa holders (SIVH) have resettled in the US annually since 2008, with the majority of applicants from Iraq and Afghanistan. SIVH include those who were employed by or on behalf of the US government and can include their families. The SIVH category from Iraq and Afghanistan includes 2 separate visa classes: Iraqi and Afghan translators/interpreters who worked for the US military and Iraqis and Afghans who worked for or on behalf of the US government [2]. SIVH qualify for many of the same benefits as refugees under the US Refugee Admissions Program (USRAP), which may include resettlement and healthcare benefits [3,4,5, 6]. The USRAP health benefits for refugees and SIVH include short-term health insurance in the US for up to 8 months and a domestic medical examination within 90 days of arrival to the US [6]. SIV admissions have been growing over the past few years, and almost 80,000 SIVH elected to use USRAP benefits from 2008 to 2018 [7].

Per the Immigration and Nationality Act, SIVH, like other immigrants and refugees, must be examined by a physician before arriving in the US. These physicians are selected by US Department of State (DOS) consular officials and must conduct the overseas medical examination in accordance with the Technical Instructions provided by the Centers for Disease Control and Prevention’s (CDC) Division of Global Migration and Quarantine [8,9]. The purpose of this examination is to determine whether an applicant has health-related grounds for inadmissibility (i.e., Class A conditions, which are conditions in which an applicant has a physical or mental disorder such as a communicable disease of public health significance that renders him or her ineligible for a visa). The results of the overseas medical examinations are noted on DOS medical forms and transmitted by the CDC to US state and local health departments via CDC’s Electronic Disease Notification system (EDN).

Despite the increase in SIV admissions to the US over recent years, little is known about the health conditions in SIV populations. According to CDC’s Guidelines for the US Domestic Medical Examination for Newly Arriving Refugees, each domestic medical examination should include a review of the overseas DOS medical forms, a complete medical history, a physical examination, screening for health conditions such as viral hepatitis and tuberculosis (TB), and the provision of preventive health interventions such as immunizations [10]. Increasing provider knowledge about the health conditions most commonly encountered in SIVH, as well as any differences in health conditions between SIVH from Iraq and Afghanistan, may facilitate diagnostic screening, examination, and referrals to additional healthcare providers in the US.

Therefore, using overseas medical examination data of US-bound SIV adults, we sought to 1) assess basic demographic characteristics of the SIV Iraqi and Afghan adult populations, 2) report the frequency and proportion of selected communicable and self-reported noncommunicable diseases (NCDs) identified during the overseas medical examinations in US-bound SIV Iraqi and Afghan adults, and 3) assess any differences in selected diseases between SIVH from Iraq and Afghanistan.

## Methods

### Participants and data collection

SIV Iraqi and Afghan adults aged ≥18 years who resettled to the US from April 2009 through December 2017 were included in this cross-sectional analysis. All data used for this analysis were collected as part of the routine overseas medical examination. Two data sources were used: 1) EDN and 2) the Worldwide Refugee Admissions Processing System (WRAPS) database holding information about native language. EDN is a centralized reporting system that notifies US state and local health departments and screening clinics of the arrival of immigrants with health conditions requiring medical follow-up, including TB-related conditions, and all refugees. A copy of health records may be also collected upon arrival at US airports by CDC quarantine station staff and data sent to EDN; these can include health records from SIVH who may not have had health conditions requiring medical follow-up identified overseas. A CDC human subjects advisor determined this project to be nonresearch; institutional review board review was not required.

We identified 36,398 Afghan and Iraqi SIVH who arrived in the US between April 2009 and December 2017 and had medical examination data recorded in EDN. We excluded 17,231 records that included SIV children <18 years of age or for whom the nationality was unable to be determined, leaving a final analytic sample of 19,167 SIV adults.

During the overseas examination, a number of tests are conducted to identify Class A (inadmissible conditions) or Class B (nonexcludable but significant physical or mental disorder) conditions, and results are conveyed via EDN. All SIV adults get a chest X-ray and, if indicated, sputum smears and cultures to assist with TB classification (see paragraph below). Adults also have a lab test for syphilis and gonorrhea. NCD histories are elicited by self-report. As mentioned earlier, all results of the overseas medical examination are collected on DOS medical forms and transmitted by the CDC to US state and local health departments via EDN.

EDN variables included were age, sex, measured height and weight, current or former tobacco use, history of illness or injury requiring hospitalization, and communicable diseases and NCDs. The disease category included any Class A or B condition and subtype of Class B TB conditions (Class B1: applicants with signs or symptoms, physical exam, or chest X-ray findings suggestive of TB disease or known HIV infection but negative sputum cultures; Class B2: applicants with a positive interferon gamma release assay [IGRA] or tuberculin skin test [TST] but otherwise a negative evaluation for tuberculosis; Class B3: applicants who recently had contact with a known infectious tuberculosis patient). Unless a waiver is granted, SIVH who are Class A for a condition at the time of the exam must address the condition before travel to the US. Once it is addressed, the examination record is updated to show resolution of the Class A status. Body mass index (BMI; kg/m^2^) was categorized as underweight (<18.5), normal range (18.5–24.99), overweight (25–29.99), and obese (≥30). Presence of NCDs was categorized as either “yes” or “no.” Current or former tobacco use was categorized as “current or former” or “never” tobacco user.

### Analyses

The variables included in this analysis and described above were outlined in a protocol used for ethical determination. We also assessed the quality and availability of our data and identified descriptive analyses appropriate for our data. Frequencies and proportions were calculated to describe the demographic characteristics and disease prevalence for all SIVH and separately for Iraqi and Afghan SIV populations.

Polytomous logistic regression was used to assess the associations of TB and BMI with nationality (Iraqi versus Afghan SIVH). For TB, the model was adjusted for age, sex, and tobacco use. For other medical conditions, we used logistic regression to assess association with nationality after adjusting for age, sex, BMI, and tobacco use. Medical conditions with 5 or fewer cases were excluded from the logistic regression analyses. A *p*-value of <0.05 was defined as statistically significant. Denominators used to estimate the prevalence of medical conditions varied because of missing data. All statistical analyses were performed with Statistical Analysis Software (version 9.3, 2011, SAS Institute, Cary, NC, USA).

## Results

Of the 19,167 SIVH, 6,813 (35.5%) were Iraqi and 12,354 (64.5%) were Afghan (Table 1). Most SIVH were male (Iraqi: 59.7%; Afghan: 54.7%) and aged 18–44 (Iraqi: 86.3%; Afghan: 95.6%). The primary languages spoken by Iraqis were Arabic (85.2%) and Kurdi (11.6%), and by Afghans, Dari (62.7%) and Pashto (35.0%).

**Table 1:** Demographic characteristics among adults ages 18 and older who are SIV applicants, EDN 2009–2017 (N = 19,167)^a^.

Demographic	SIV—All	SIV—Iraq	SIV—Afghanistan
	N (%)	N (%)	N (%)
**Total**	19,167	6,813 (35.5%)	12,354 (64.5%)
**Sex**			
Female	8,367 (43.7%)	2,775 (40.7%)	5,592 (45.3%)
Male	10,800 (56.3%)	4,038 (59.7%)	6,762 (54.7%)
**Age**			
18–44	17,693 (92.3%)	5,881 (86.3%)	11,812 (95.6%)
45–64	1,413 (7.4%)	887 (13.0%)	526 (4.2%)
65 and older	61 (0.3%)	45 (0.7%)	16 (0.1%)
**Native language**^b^	*n* = 19,087	*n* = 6,810	*n* = 12,277
Dari		–	7,700 (62.7%)
Pashto		–	4,294 (35.0%)
Arabic		5,802 (85.2%)	
Kurdi		790 (11.6%)	–
Others		218 (3.2%)	283 (2.3%)

^a^Percentages may not add up to 100% because of rounding.

^b^EDN data linked with WRAPS database with a final analytic sample of 19,087 SIVH.

**Abbreviations:** EDN, Electronic Disease Notification system; SIV, Special Immigrant Visa; SIVH, SIV holder; WRAPS, Worldwide Refugee Admissions Processing System.

Overall, 316 of 19,167 (1.6%) SIVH were reported to have a Class B TB condition (Table 2). In the adjusted analysis, Iraqis were less likely than Afghans to have Class B1 TB (odds ratio [OR]: 0.2; 95% confidence interval [CI]: 0.1–0.3) or B2 TB (OR: 0.2; 95% CI: 0.1–0.3). Six SIVH had historical evidence of Class A TB diagnosis and treatment or were on treatment at the time of the overseas medical examination; 5 were Afghan, and 1 was Iraqi. There were no reports of gonorrhea (0%), Hansen’s disease (0%), or remission of addiction (0%) from the overseas medical examination for SIVH. Approximately 19.4% of all SIVH self-reported as current or former smokers (N = 3,543) (Table 3), with 28.8% of Iraqis and 14.2% of Afghans being current or former smokers. The leading self-reported NCDs among all SIVH from our data included overweight/obesity (56.5%), hypertension (2.4%), diabetes (1.1%), and thyroid disorder (0.7%) (Table 3). Approximately 30.8% of all SIVH reported a history of illness or injury that required hospitalization. In the adjusted analysis, Iraqi SIVH were 2 to 7 times more likely than Afghan SIVH to have reported NCDs. More specifically, Iraqis were 3.7 times more likely to have obesity (95% CI: 3.4–4.0), 2.5 times more likely to have diabetes (95% CI: 1.7–3.5), and 3.7 times more likely to have a thyroid disorder (95% CI: 2.4–5.8). Iraqis were 2.5 times more likely to be a current or former smoker compared to Afghans (95% CI: 2.3–2.7).

**Table 2:** Prevalence of Class B conditions among adults ages 18 and older who are SIV applicants, EDN 2009–2017 (N = 19,167)^a^.

Class B Conditions	SIV—All	SIV—Iraq	SIV—Afghanistan	Adjusted OR (95% CI)
	N (%)	N (%)	N (%)	Presence of Health Condition
**Total**	19,167	6,813 (35.5%)	12,354 (64.5%)	
**TB**^b^				
No TB classification	18,851 (98.4%)	6,777 (99.5%)	12,074 (97.7%)	Reference
Pulmonary/extrapulmonary^c^ (Class B1)	156 (0.8%)	20 (0.3%)	136 (1.1%)	0.2 (0.1, 0.3)
LTBI^d^ (Class B2)	159 (0.8%)	16 (0.2%)	143 (1.2%)	0.2 (0.1, 0.3)
Contact^e^ (Class B3)	1 (0.01%)	0 (0%)	1 (0.01%)	–
**Syphilis**^f^	27 (0.1%)	5 (0.03%)	22 (0.2%)	

^a^Percentages may not add up to 100% because of rounding.

^b^Polytomous logistic regression model, adjusted for age, sex, and tobacco use was used to assess the association between TB and nationality (Iraqi or Afghan; Afghan origin as reference).

^c^Class B1: Applicants with signs or symptoms, physical exam, or chest X-ray findings suggestive of TB disease or known HIV infection but negative sputum cultures. Among 112 SIVH (out of 156) with data available on type of Class B1 TB, 111 had pulmonary TB and 1 had extrapulmonary TB.

^d^Class B2 or LTBI: Applicants with a positive IGRA or TST but otherwise a negative evaluation for tuberculosis.

^e^Class B3: Applicants who recently had contact with a known infectious TB patient.

^f^Syphilis is considered Class B if treated overseas.

**Abbreviations:** CI, confidence interval; EDN, Electronic Disease Notification system; IGRA, interferon gamma release assay; LTBI, latent tuberculosis infection; OR, odds ratio; SIV, Special Immigrant Visa; SIVH, SIV holder; TB, tuberculosis; TST, tuberculin skin test.

**Table 3:** Prevalence of overweight/obesity, tobacco use, and medical conditions among adults ages 18 and older who are SIV applicants, EDN 2009–2017 (N = 19,167)^a^.

	SIV—All	SIV—Iraq	SIV—Afghanistan	Adjusted OR
	N (%)	N (%)	N (%)	Presence of Risk Factor/Health Condition
**Total**	19,167	6,813 (35.5%)	12,354 (64.5%)	
**BMI (kg/m**^**2**^**)**^b^ **(*n* = 17,870)**				
Underweight <18.5	516 (2.9%)	75 (1.2%)	441 (3.8%)	0.5 (0.4, 0.7)
Normal range 18.5–24.99	7,260 (40.6%)	1,770 (28.3%)	5,490 (47.3%)	Reference
Overweight 25–29.99	6,740 (37.7%)	2,539 (40.5%)	4,201 (36.2%)	1.8 (1.7, 1.9)
Obese ≥30	3,354 (18.8%)	1,881 (30.0%)	1,473 (12.7%)	3.7 (3.4, 4.0)
**Tobacco use (*n* = 18,272)**				
Current or former	3,543 (19.4%)	1,869 (28.8%)	1,674 (14.2%)	2.5 (2.3, 2.7)
Current	2,660 (76.1%)	1,458 (79.8%)	1,202 (72.1%)	
Former	835 (23.4%)	370 (20.2%)	465 (27.9%)	
Never	14,740 (80.6%)	4,610 (71.2%)	10,130 (85.8%)	
**Medical conditions**^c^				
Anemia (*n* = 10,593)	10 (0.1%)	7 (0.4%)	3 (0.03%)	NA
Asthma (*n* = 18,272)	71 (0.4%)	57 (0.9%)	14 (0.1%)	6.5 (3.5, 12.1)
Cardiac condition^d^ (*n* = 19,156)	67 (0.4%)	48 (0.7%)	19 (0.2%)	2.9 (1.6, 5.2)
Chronic hepatitis (*n* = 18,274)	46 (0.3%)	4 (0.06%)	42 (0.4%)	NA
Chronic renal disease (*n* = 18,274)	26 (0.1%)	15 (0.2%)	11 (0.1%)	2.4 (1.0, 5.5)
Diabetes (*n* = 18,274)	194 (1.1%)	144 (2.2%)	50 (0.4%)	2.5 (1.7, 3.5)
Hypertension (*n* = 18,273)	432 (2.4%)	244 (3.8%)	188 (1.6%)	0.9 (0.7, 1.1)
Major mental disorder (*n* = 18,272)	16 (0.09%)	4 (0.06%)	12 (0.1%)	NA
Malignancy (*n* = 18,282)	14 (0.08%)	5 (0.08%)	9 (0.08%)	NA
Seizure disorder (*n* = 18,271)	27 (0.1%)	25 (0.4%)	2 (0.02%)	NA
Thyroid disorder (*n* = 18,281)	121 (0.7%)	87 (1.3%)	34 (0.3%)	3.7 (2.4, 5.7)
**Illness or injury requiring hospitalization (*n* = 18,269)**	5,634 (30.8%)	2,693 (41.6%)	2,941 (24.9%)	2.1 (2.0, 2.3)

^a^Percentages may not add up to 100% because of rounding. A *p*-value of <0.05 was considered statistically significant.

^b^Polytomous logistic regression model, adjusted for age and sex, was used to assess the association between BMI and nationality (Iraqi or Afghan; Afghan origin as reference).

^c^Multivariable logistic regression models were fitted to estimate adjusted ORs and 95% CIs for health conditions when there were >5 counts per cell. Multivariable logistic regression models included the health condition as the outcome variable; SIV origin (Iraqi or Afghan; Afghan origin as reference) as the primary exposure variable; and age, sex, BMI, and tobacco use as the covariates.

^d^Examples of cardiac conditions include congenital heart disease, heart valve disease, or ischemic heart disease.

**Abbreviations:** BMI, body mass index; CI, confidence interval; EDN, Electronic Disease Notification system; OR, odds ratio; SIV, Special Immigrant Visa.

## Discussion

Among adult Iraqi and Afghan SIVH who resettled to the US between 2009 and 2017, most were men aged 18 to 44 years. Only 1.6% of all SIV adults were reported to have a Class B TB condition, and Iraqi SIVH were less likely to have a Class B TB condition than were Afghan SIVH. Overweight/obesity, hypertension, diabetes, and thyroid disorder were the most common NCD conditions self-reported among all SIVH. About one in seven SIV adults were current smokers. Compared to Afghan SIVH, Iraqi SIVH had a higher likelihood of being overweight or having obesity, having diabetes, or having a thyroid disorder and were more likely to be a current or former smoker.

While our study reports a low percentage of SIV adults identified with a TB condition from the overseas examination, other studies suggest that TB may be more common in Iraqi and Afghan populations. TB infection prevalence estimates in the Afghan population are around 15% [11]. Further, statistics from the World Health Organization (WHO) note that in 2017, the incidence of TB was 42 per 100,000 people in Iraq and 189 per 100,000 people in Afghanistan [12,13]. Given that few SIVH had active TB disease at the time of the overseas exam in this analysis, it is possible that the SIV population may have less TB risk factor exposure than the general Iraqi and Afghan populations.

While data on latent TB infection prevalence among Afghan populations are minimal to absent in the literature, latent TB infection prevalence estimates in Iraqi populations range from 0.9% to 14.1% [14,15]. Of note, overseas medical examinations are not required to screen for latent TB infection [16]. Regardless, CDC’s Guidelines for Screening for Tuberculosis Infection and Disease during the Domestic Medical Examination for Newly Arrived Refugees ensure that SIVH who elect to use health benefits and arrive for their domestic medical examination are routinely reevaluated for TB by US clinicians and offered appropriate treatment if needed [17]. This reevaluation includes testing adults with IGRA or TST to document latent TB infection if results from these tests are not available from the overseas examination.

The finding that the leading self-reported NCDs in this analysis included overweight/obesity, hypertension, and diabetes is supported by other studies of Afghan and Iraqi immigrants and refugees in the US and other countries, including immigrants and refugees who were tested either overseas or soon after arrival in the resettlement country [12–15,18–21]. In our investigation, with the exception of overweight and obesity, the proportions of self-reported NCDs are substantially lower in both SIV populations than in other analyses; e.g., estimates of hypertension or high blood pressure ranged from 21% to 28% among Afghan populations [12,18,19] and 10% to 33% among Iraqi populations [13–15,20,21]. The reasons for these discrepancies could include fear that disclosure of any medical conditions may prohibit travel to and resettlement in the US. Further, clinical or laboratory testing is not required for these conditions before US resettlement. SIVH may also be unaware that they have these medical conditions. Lastly, it is also possible that access to healthcare and preventive health services is limited for SIV populations overseas [22]; therefore, these conditions could be underdiagnosed. However, chronic diseases are commonly reported longer-term health outcomes among immigrant populations in the US [23,24], although these data are not currently available for SIV populations. Therefore, clinicians should consider screening and evaluating for certain NCDs based on age and risk factors in the early resettlement period, especially given potential barriers (e.g., language, health literacy, navigating US healthcare system) to healthcare access encountered by immigrant populations.

Only 2.2% of Iraqi SIV adults in our investigation reported having diabetes, which is lower than the diabetes prevalence found in other studies among Iraqi populations, which range from 2.7% to 11% [13,15,20,21]. However, Bennet and colleagues reported that a third of Iraqi adult study participants with normal waist circumferences (versus 9% of Swedish counterparts) and 56% of obese Iraqis (versus 42%) were insulin-resistant, suggesting the possibility of an irregular fat metabolism in Iraqis (specifically males) [25]. Diabetes prevalence among Afghan adults ranges between 9% and 11%, again higher than reported in our investigation [12,19]. Given that both refugees and immigrants face an increased risk for developing diabetes after resettlement in the United States compared to nonimmigrant US residents [26] and encounter significant barriers to use of the US healthcare system [27,28], US clinicians who care for SIVH during the domestic medical examination should consider screening for diabetes among SIV adults with risk factors. Such screening may prompt earlier diagnosis and management.

Among SIV populations, a higher proportion are current smokers than former smokers before arrival into the US. Other studies also reveal that smoking is common among Iraqi adults (18%–29%) [15,29,30] and Afghan adults (14%) [19]. Therefore, providers who screen and care for SIVH upon entry into the US should consider screening for smoking and promptly initiating or referring SIVH to smoking cessation counseling and resources.

Another key finding from our investigation is that Iraqi SIVH, compared to Afghan SIVH, have higher proportions of obesity, diabetes, or a thyroid disorder or were more likely to be a current or former smoker. Few studies have compared the health profiles of these 2 populations. However, the 2013 Global Burden of Disease Study reported a higher percentage of disability-adjusted life years attributed to high BMI, high fasting blood glucose, or smoking among the Iraqi population than among the Afghan population, although the statistical significance of these differences was not reported [31].

Our analysis has several limitations. First, health information for all SIVH is not available for analysis, for several reasons. Medical information for SIVH until recently (beginning in 2017) was included in EDN only if the SIVH had a Class A or B health condition. While it is possible that a copy of health records was collected upon arrival at US airports by CDC quarantine station staff and data sent to EDN, this likely happened inconsistently. Therefore, our findings cannot be generalized to all SIV populations entering the US. Second, although CDC’s Technical Instructions state that a medical history including several NCDs should be noted during the overseas medical examination, physicians are not required to investigate or conduct laboratory testing to diagnose NCDs. Thus, a full diagnostic workup for these conditions (e.g., fasting blood glucose or hemoglobin A1c, thyroid-stimulating hormone, complete blood cell count) is not conducted before travel to the US. Access to healthcare and preventive health services may also be limited overseas. Therefore, a number of these health conditions could be underreported, underdiagnosed, or both, and our estimates for NCDs likely do not reflect the true burden of NCDs among SIV populations. Third, given absent data on SIV populations in the literature, the authors must consider comparing estimates of disease among SIVH reported in this analysis with disease estimates of other populations of similar nationality (e.g., refugee and other immigrant populations). Given differing historical, socioeconomic, and migration backgrounds between these populations, these comparisons may not always be appropriate. Lastly, estimates of NCDs may be underestimated or overestimated because of missing data for several NCDs.

To the authors’ knowledge, this is the first analysis assessing proportions of selected communicable diseases and NCDs among US-bound adult Iraqi and Afghan SIV populations. In addition to following the recommendations in CDC’s Guidelines for the US Domestic Medical Examination for Newly Arriving Refugees, state public health agencies and clinicians screening SIVH should consider screening for diabetes among those with risk factors and prompt referral and management of obesity, hypertension, and smoking [10]. Behavioral risk factor counseling and referral to culturally appropriate programs such as the Diabetes Prevention Program can be initiated at screening visits and subsequently reemphasized with primary care providers and other healthcare professionals [32]. Clinicians should also be aware that Iraqi and Afghan SIV populations differ in socioeconomic and migration histories from refugee populations from the same countries; e.g., SIVH from Iraq and Afghanistan are considered to have higher income levels and living standards compared to their respective refugee populations from the same countries. Future studies can explore the health profile of SIV populations, including a more comprehensive assessment of the prevalence of NCDs such as mental health conditions, after integration into the US.

## Abbreviations

BMI: body mass index
CDC: Centers for Disease Control and Prevention
CI: confidence interval
DOS: Department of State
EDN: Electronic Disease Notification system
IGRA: interferon gamma release assay
NCD: noncommunicable disease
OR: odds ratio
SIV: Special Immigrant Visa
SIVH: SIV holder
TB: tuberculosis
TST: tuberculin skin test
US: United States
USRAP: US Refugee Admissions Program
WHO: World Health Organization
WRAPS: Worldwide Refugee Admissions Processing System
